# Imaging Endocannabinoids and Bioactive Lipid Messengers in Basic Research and Biomedical Application

**DOI:** 10.3390/cells11111756

**Published:** 2022-05-26

**Authors:** Jun Aoki, Masako Isokawa

**Affiliations:** 1 Graduate School of Frontier Biosciences, Osaka University, Osaka 565-0871, Japan; junaoki@fbs.osaka-u.ac.jp; 2 Laboratory for Cell Signaling Dynamics, RIKEN Center for Biosystems Dynamics Research, Saitama 351-0198, Japan; 3 Forefront Research Center, Graduate School of Science, Osaka University, Osaka 599-8531, Japan

Endocannabinoids (eCBs) are representative bioactive lipid messengers. These lipids are indispensable in our lives, playing critical roles in development, learning and memory. For example, in the brain, bioactive lipids and metabolites are important constituents of central neuron synapses. Lipid rafts are fundamental in postsynaptic domains, and eCBs regulate neurotransmitter release at presynaptic terminals. Although super-resolution fluorescent imaging techniques such as PALM, STORM and STED microscopy have visualized single proteins and nucleic acids, and advanced our knowledge of synaptic structure and function, we cannot utilize this technique for lipid imaging because lipids cannot be tagged easily with fluorescent probes, or engineered genetically in order to possess fluorescing components. The majority of lipids remain unseen without visualization in spite of their importance in life sciences.

In this Special Issue, we discuss technologies that enable us to see eCBs and bioactive lipid messengers such as *fatty acids and lyso-phospholipids* directly on biological specimens, just like seeing them under the microscope. This approach not only opens up a new strategy in lipidomic research, but also provides a fuller understanding of lipid function and metabolic pathways in health and disease. Our goal is to unveil the cytoplasmic and cytosolic localization of eCBs and bioactive lipids with integrated cell morphology, by utilizing the right tools. 

The imaging mass spectrometry (IMS) of MALDI, ESI, DESI, and SIMS has allowed the rapid development of cutting-edge techniques for measuring and mapping the localization of signaling lipids and metabolites, by ionizing them directly in label-free conditions [1]. In order to visualize individual lipid molecules, the high-resolution single-cell imaging technique, which comprises a new laser assembly and novel ion optics, has emerged to complement the insufficient spatial resolution typically found in conventional IMS equipment [2]. In addition, Raman microscopy [3], vibrational spectroscopy [4], and lipophilic fluorescent indicators [5] make unique contributions in locating eCBs and other signaling lipids. We hope you will enjoy reading the collection of excellent papers on these topics presented in this Special Issue. We wish for this Special Issue to be an informative forum for both biologists and physicists who are interested in developing innovative new technologies for visualizing eCBs and bioactive lipids at sub-cellular resolution.

## References

[1] Iwama T., Kano K., Saigusa D., Ekroos K., van Echten-Deckert G., Vogt J., Aoki J. (2021). Development of an on-tissue derivatization method for MALDI mass spectrometry imaging of bioactive lipids containing phosphate monoester using phos-tag. Anal. Chem..

[2] Aoki J., Toyoda M. (2021). Development of novel projection-type imaging mass spectrometer. Rev. Sci. Instrum..

[3] Ando J., Kinoshita M., Cui J., Yamakoshi H., Dodo K., Fujita K., Murata M., Sodeoka M. (2015). Sphingomyelin distribution in lipid rafts of artificial monolayer membranes visualized by Raman microscopy. Proc. Natl. Acad. Sci. USA.

[4] Cheng J.-X., Xie X.S. (2015). Vibrational spectroscopic imaging of living systems: An emerging platform for biology and medicine. Science.

[5] Dong A., He K., Dudok B., Farrell J.S., Guan W., Liput D.J., Puhl H.L., Cai R., Wang H., Duan J. (2021). A fluorescent sensor for spatiotemporally resolved imaging of endocannabinoid dynamics in vivo. Nat. Biotechnol..

